# Comparison of Serological Response to Doxycycline versus Benzathine Penicillin G in the Treatment of Early Syphilis in HIV-Infected Patients: A Multi-Center Observational Study

**DOI:** 10.1371/journal.pone.0109813

**Published:** 2014-10-13

**Authors:** Jen-Chih Tsai, Yu-Huei Lin, Po-Liang Lu, Ni-Jiin Shen, Chia-Jui Yang, Nan-Yao Lee, Hung-Jen Tang, Yuag-Meng Liu, Wen-Chi Huang, Chen-Hsiang Lee, Wen-Chien Ko, Yen-Hsu Chen, Hsi-Hsun Lin, Tun-Chieh Chen, Chien-Ching Hung

**Affiliations:** 1 Department of Internal Medicine, National Taiwan University Hospital and National Taiwan University College of Medicine, Taipei, Taiwan; 2 Department of Internal Medicine, Tzu-Chi Hospital and Tzu-Chi University College of Medicine, Hua-Lien, Taiwan; 3 Department of Internal Medicine, Taichung Veterans General Hospital, Taichung, Taiwan; 4 Department of Internal Medicine, Kaohsiung Medical University Hospital and College of Medicine, Kaohsiung Medical University, Kaohsiung, Taiwan; 5 Department of Internal Medicine, National Taiwan University Hospital Yun-Lin Branch, Yun-Lin, Taiwan; 6 Department of Internal Medicine, Far Eastern Memorial Hospital, New Taipei City, Taiwan; 7 Department of Internal Medicine, National Cheng Kung University College of Medicine and Hospital, Tainan, Taiwan; 8 Department of Internal Medicine, Chi Mei Medical Center, Tainan, Taiwan; 9 Department of Internal Medicine, Changhua Christian Hospital, Changhua, Taiwan; 10 Department of Internal Medicine, Kaohsiung Chang Gung Memorial Hospital, Chang Gung University College of Medicine, Kaohsiung, Taiwan; 11 Department of Internal Medicine, E-Da Hospital/I-Shou University, Kaohsiung, Taiwan; 12 Department of Internal Medicine, Kaohsiung Municipal Ta-Tung Hospital, Kaohsiung Medical University, Kaohsiung, Taiwan; 13 Department of Medical Research, China Medical University Hospital, Taichung, Taiwan; 14 China Medical University, Taichung, Taiwan; University Hospital Zurich, Switzerland

## Abstract

**Background:**

While doxycycline is recommended as an alternative treatment of syphilis in patients with penicillin allergy or intolerance, clinical studies to compare serological response to doxycycline versus benzathine penicillin in treatment of early syphilis among HIV-infected patients remain sparse.

**Methods:**

We retrospectively reviewed the medical records of HIV-infected patients with early syphilis who received doxycycline 100 mg twice daily for 14 days (doxycycline group) and those who received 1 dose of benzathine penicillin (2.4 million units) (penicillin group) between 2007 and 2013. Serological responses defined as a decline of rapid plasma reagin titer by 4-fold or greater at 6 and 12 months of treatment were compared between the two groups.

**Results:**

During the study period, 123 and 271 patients in the doxycycline and penicillin group, respectively, completed 6 months or longer follow-up. Ninety-one and 271 patients in the doxycycline and penicillin group, respectively, completed 12 months or longer follow-up. Clinical characteristics were similar between the two groups, except that, compared with penicillin group, doxycycline group had a lower proportion of patients with secondary syphilis (65.4% versus 41.5%, *P*<0.0001) and a higher proportion of patients with early latent syphilis (25.3% versus 49.6%, *P*<0.0001). No statistically significant differences were found in the serological response rates to doxycycline versus benzathine penicillin at 6 months (63.4% versus 72.3%, *P* = 0.075) and 12 months of treatment (65.9% versus 68.3%, *P* = 0.681). In multivariate analysis, secondary syphilis, but not treatment regimen, was consistently associated with serological response at 6 and 12 months of follow-up.

**Conclusions:**

The serological response rates to a 14-day course of doxycycline and a single dose of benzathine penicillin were similar in HIV-infected patients with early syphilis at 6 and 12 months of follow-up. Patients with secondary syphilis were more likely to achieve serological response than those with other stages.

## Introduction

According to the WHO estimate, there were 36.4 million cases of syphilis globally in adults aged between 15 and 49 years in 2008 [1]. In the United Kingdom, the newly diagnosed cases of syphilis increased from 2,650 in 2010 to 2,915 in 2011 and 75% of the cases occurred in men who have sex with men (MSM) [2]. In the United States, the case number of syphilis has been increasing since 2000, and most of the cases also occur in MSM, many of whom are coinfected with HIV [3]. The increasing rates of syphilis among MSM have raised concerns about the mutually detrimental interactions between HIV infection and syphilis and several studies have shown that syphilis is associated with increased risks of transmission of HIV and hepatitis viruses [4]–[9]. HIV-infected patients with primary syphilis may present with more ulcers at the inoculated sites [10] and neurological complications [11], [12]; furthermore, HIV-infected patients with syphilis are at higher risk of serological failure despite appropriate treatments [13], although not all studies share the same finding [14].

According to the treatment guidelines for sexually transmitted diseases (STDs) by the Centers for Disease Control and Prevention (CDC) [15] and UK national guidelines on the management of syphilis [16], benzathine penicillin is the treatment of the choice for syphilis and one dose of benzathine penicillin (2.4 million units [MU]) administered intramuscularly is recommended for HIV-infected and HIV-uninfected patients with early syphilis (primary, secondary, and early latent syphilis). If the patients are allergic to penicillin, doxycycline is the preferred alternative agent at a dose of 100 mg twice daily for 14 days for early syphilis and 28 days for late latent syphilis or syphilis of unknown duration. The serological response rate to doxycycline in the previous retrospective studies conducted in the era of HIV epidemic ranged from 73.3% to 100% [17]–[20]. However, the numbers of HIV-infected patients with syphilis enrolled to receive doxycycline in these studies were small, ranging from 0 to 15 [17]–[19] and definitions of serological response and time points to determine the response were different. In this study, we aimed to compare the serological response rates of early syphilis to doxycycline versus benzathine penicillin among HIV-infected patients by following the STDs treatment guidelines [15], [16], [21].

## Methods

### Study population and setting

This multicenter, retrospective study was conducted by infectious diseases specialists at 9 hospitals designated for HIV care around Taiwan, where inpatient or outpatient HIV care, including combination antiretroviral therapy (cART), treatments of HIV-related opportunistic illnesses, and monitoring of plasma HIV RNA load (PVL) and CD4 lymphocyte counts, are reimbursed by the government. According to the national guidelines for management of HIV-infected patients, non-treponemal tests for syphilis are recommended to be performed every 6 to 12 months for those who do not have syphilis and every 3 to 6 months over a period of 2 years for those with treated syphilis. The treatment regimens for syphilis were prescribed according to the STDs treatment guidelines of US CDC in 2006 and 2010 [15], [21].

We reviewed the medical records of the HIV-infected patients aged 20 years or greater, who presented with early syphilis and received a 14-day treatment course of doxycycline or a single dose of benzathine penicillin (2.4 MU) at the participating hospitals from January 2007 to August 2013. Patients were excluded from analysis, if antibiotics were concurrently given that were treatment options for syphilis such as ceftriaxone or azithromycin when early syphilis was diagnosed; or if those antibiotics were used for treatment of diseases other than syphilis during the 6 months of follow-up after doxycycline or benzathine penicillin treatment. Patients with RPR titers of less than 4 was not included because of concerns about increased risk of biological false-positive syphilis serologies (RPR titer of 1∶1 or 1∶2) [22]. Neurosyphilis such as CNS dysfunction, stroke, auditory and ophthalmic abnormalities or tertiary syphilis were also excluded. The study was approved by the Research Ethics Committees of the participating hospitals (National Taiwan University Hospital [20l003111R and 201303017RINB], Kaohsiung Medical University Hospital [KMUH-IRB-20110040], Kaohsiung Municipal Ta-Tung Hospital [KMUHIRB-20130016 and KMUHIRB-20130017], Far Eastern Memorial Hospital [FEMH-IRB-099023-F], Kaohsiung Chang Gung Memorial Hospital [99-1934B], E-Da Hospital [EMRP-099-106], National Cheng Kung University Hospital [BR-99-078-C and A-BR-103-003], Chi Mei Medical Center [10306-007], and Changhua Christian Hospital [140608]) and written or oral informed consent was waived. The data we collected for this paper will be made fully available without restriction after decoding and approval by the respective Research Ethics Committee of each participating hospital.

The outcomes of interest were serological response rates at 6 and 12 months of treatment. We also examined the reasons of failure to achieve serological response at 6 and 12 months of treatment in the two groups (non-responders).

### Data collection

A standardized case record form was used to collect information on demographic characteristics, risk behavior for HIV transmission, PVL and CD4 counts at baseline and during follow-up, cART, stage of syphilis, RPR titer before treatment and during the first 6 and 12 months of follow-up, and the first episode of recurrent syphilis and its stage.

### Laboratory investigations

Serological tests for syphilis were performed with the use of rapid RPR test (BD Macro-VueTMRPR Card tests, USA) and *Treponema pallidum* hemagglutination test (FTI-SERODIA-TPPA. Fujirebio Taiwan Inc., Taoyuan, Taiwan) in the participating hospitals. Plasma HIV RNA load and CD4 lymphocyte count were quantified by the Cobas Amplicor HIV-1 Monitor™ Test, version 1.5, (Roche Diagnostics Corporation, Indianapolis, USA) and FACSFlow (Becton Dickinson), respectively.

### Definitions

Early syphilis that includes primary, secondary and early latent syphilis was defined according to STDs treatment guidelines of US CDC in 2010 [15]. Patients were diagnosed as having primary syphilis if they had ulcers or chancre at the infection site; secondary syphilis if they developed skin rash, mucocutaneous lesions, or lymphadenopathy in the presence of seroreactivity; and early latent syphilis if they acquired syphilis within the preceding year that was characterized by seroreactivity without clinical manifestations. Serological response was defined as a decline of RPR titer by 4-fold or greater from the baseline value at 6 or 12 months of doxycycline or benzathine penicillin treatment. The last-observed-carried-forward principle was used to deal with missing values of RPR titers at 6 months and 12 months following treatment. Non-responders were those who received another course of treatment regardless of serological response during the follow-up; or those who failed to achieve a decline of RPR titers by 4-fold or greater at 6 months and 12 months following treatment. Failure to achieve serological response could be caused by treatment failure or reinfection with syphilis. In this study, a diagnosis of reinfection was made in patients who developed new symptoms of primary or secondary syphilis; or those who had an increase of RPR titer by 4-fold or greater after ever achieving serological response during follow-up, while treatment failure was defined as failure to achieve a decline of RPR titers by 4-fold or greater or receipt of another course of treatment without demonstrated serological response throughout the follow-up period.

### Statistical Analysis

All statistical analyses were performed using SPSS software version 17.0 (SPSS Inc., Chicago, IL). Categorical variables were compared using χ^2^ or Fisher's exact test whereas non-categorical variables were compared using Student's *t* test or Mann-Whitney *U* test. All tests were two-tailed and a *P* value <0.05 was considered significant. Multiple logistic regression method was used to identify factors associated with serological response at 6 and 12 months of treatment. We included the variables with a *P*-value <0.2 in the univariate analysis such as age, CD4 count <350 cells/µl, a history of prior syphilis, stage of syphilis and variables that were of biological significance such as antibiotic administered (doxycycline versus penicillin) in the multivariate logistic regression models.

## Results

During the study period, 123 patients who received a 14-day course of doxycycline (doxycycline group) and 271 patients who received 1-dose benzathine penicillin (penicillin group) were enrolled. All patients in the penicillin group had completed 12 months or longer follow-up, while in the doxycycline group all patients had completed 6 months and 91 had completed 12 months or longer follow-up. The demographic and clinical characteristics are shown in Table 1. All the patients were men, with a median age of 32 years (range, 20–59 years) in the doxycycline group and 31 years (range, 20–71 years) in the penicillin group. Most of the patients were MSM in either group. In the doxycycline group, more patients presented with early latent syphilis (49.6%) than secondary syphilis (41.5%), while more patients in the penicillin group presented with secondary syphilis (65.4%); however, the median RPR titer (64) was the same for both groups. There were no statistically significant differences in terms of baseline CD4 count, PVL, the proportion of PVL <400 copies/ml, and the proportion of patients on cART when the patients sought treatment of early syphilis between the two groups.

**Table 1:** Clinical characteristics of the HIV-infected patients with early syphilis who received doxycycline or benzathine penicillin.

	Doxycycline (N = 123)	Benzathine penicillin (N = 271)	*P*-value
**Male gender, n (%)**	123 (100)	271 (100)	0.99
**Age, median (range), years**	32(20–59)	31.4 (20–71)	0.768
**Risk behavior for HIV transmission, n (%)**			
**MSM**	114 (92.7)	260 (95.9)	0.172
**heterosexual**	1 (0.8)	8 (3)	0.188
**unknown**	8 (6.5)	3 (1.1)	0.003
**Stage of syphilis, n (%)**			
**primary**	11 (8.9)	24 (9.3)	0.978
**secondary yphilis**	51 (41.5)	167 (65.4)	<0.0001
**early latent**	61 (49.6)	80 (25.3)	<0.0001
**RPR titer, median**	1∶64	1∶64	0.147
**CD4 count, mean (SD), cells/µl**	450 (229)	466 (250)	0.531
**CD4 <200, n (%)**	12 (9.8)	27 (10)	0.949
**CD4 <350, n (%)**	49 (39.8)	90 (33.2)	0.202
**PVL, mean (SD), log_10_ copies/ml**	2.99 (1.54)	3.10 (1.51)	0.92
**PVL <400 copies/ml, n (%)**	66 (53.7)	142 (52.4)	0.816
**cART, n (%)**	80 (65.0)	174 (64.2)	0.873
**Prior history of syphilis, n (%)**	74 (60.2)	92 (33.9)	<0.001

**Abbreviations**: cART, combination antiretroviral therapy; MSM, men who have sex with men; PVL, plasma HIV RNA load; RPR, rapid plasma reagin; SD, standard deviation.

**Figure 1:**
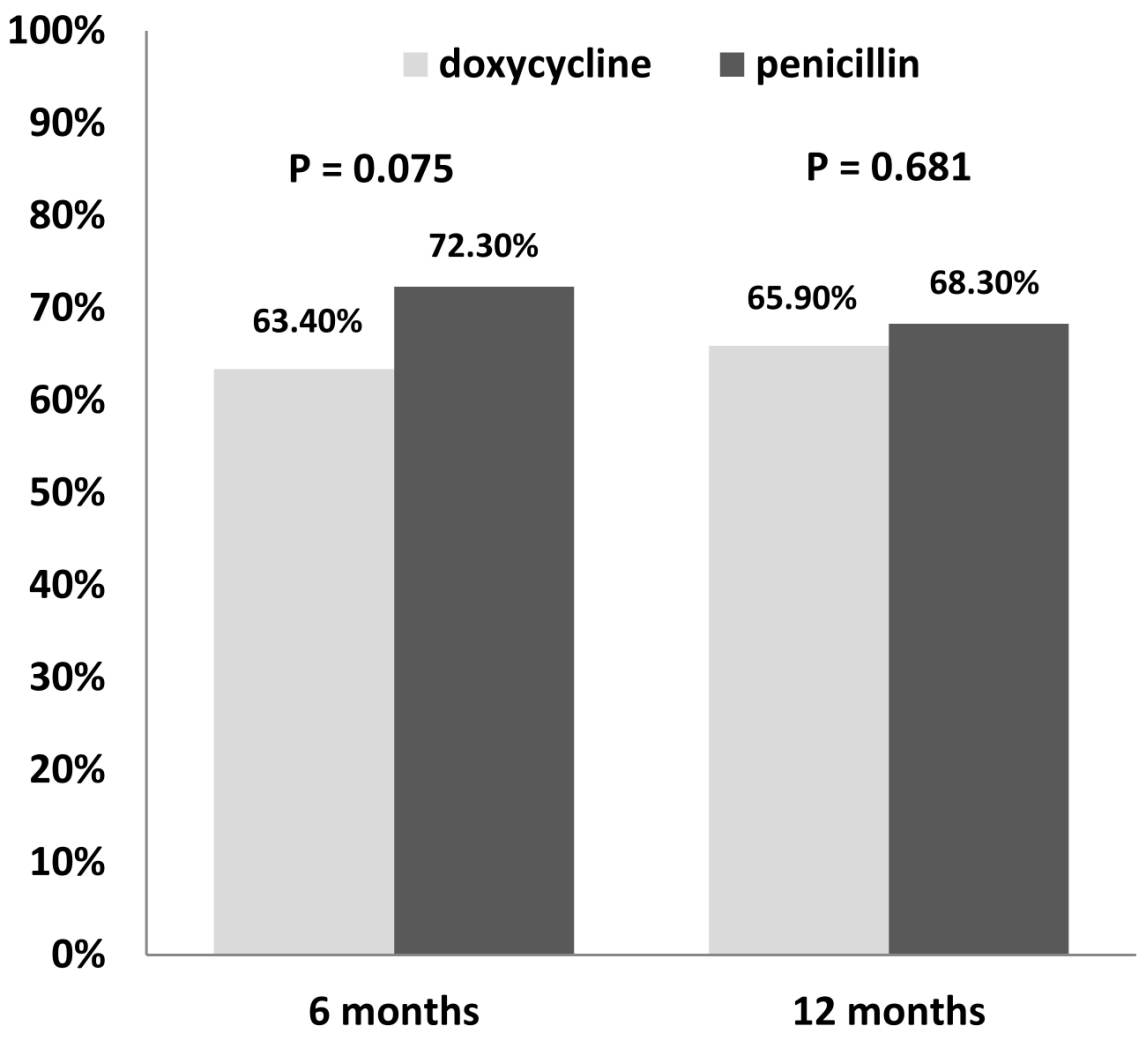
Serological response rates at 6 months and 12 months in the doxycycline group and benzathine penicillin group following treatment of early syphilis.

The serological response rate at 6 months of treatment was lower, though not statistically significant, in the doxycycline group than the penicillin group (63.4% vs 72.3%, *P* = 0.075), while at 12 months the response rates were similar between the two groups: 65.9% in the doxycycline group and 68.3% in the penicillin group (*P* = 0.681) (Fig. 1).

In univariate analysis, patients who achieved serological response at 6 months following treatment were significantly younger than those who failed to achieve serological response (32.0 vs 34.8 years, *P* = 0.001). At 6 and 12 months of follow-up, patients who presented with secondary syphilis had a higher serological response rate than those who did not present with secondary syphilis (59.9% vs 45.0% , *P* = 0.006; and 62.4% vs 44.4%, *P* = 0.001, respectively); in contrast, patients who presented with early latent syphilis had a lower response rate than those who did not present with early latent syphilis at 6 and 12 months (31.8% vs 45.0%, *P* = 0.012; and 28.6% vs 45.3%, *P* = 0.002, respectively) (Table 2). A history of prior syphilis was also associated with lower serological response rates at 6 and 12 months (38.3% vs 50.8%, *P* = 0.021; and 38.4% vs 48.7%, *P* = 0.062, respectively) (Table 2).

**Table 2:** Univariate analysis of factors associated with serological response at 6 months and 12 months following treatment of early syphilis.

	6 months			12 months		
	Response N = 274	No response N = 120	*P* =	Response N = 245	No response N = 117	*P* =
**Age, median (range), years**	30 (20–71)	34 (20–61)	0.001	31 (20–71)	33 (21–59)	0.768
**Treatment**						
**Doxycycline**	78 (28.5)	45 (37.5)	0.075	60 (24.5)	31 (26.5)	0.681
**Penicillin**	196 (71.5)	75 (62.5)		185 (75.5)	86 (73.5)	
**Stage of syphilis**						
**primary, n (%)**	23 (8.4)	12 (10.0)	0.606	22 (9.0)	12 (10.3)	0.697
**secondary**	164 (59.9)	54 (45.0)	0.006	153 (62.4)	52 (44.4)	0.001
**early latent**	87 (31.8)	54 (45.0)	0.012	70 (28.6)	53 (45.3)	0.002
**Risk for HIV transmission, n (%)**						
**MSM**	262 (95.6)	112 (93.3)	0.341	232 (94.7)	111 (94.9)	0.943
**CD4 count, mean (SD), cells/µl**	468.2 (223.8)	445.0 (259.5)	0.148	460.7 (231.1)	470.3 (251.2)	0.531
**CD4 <200, n (%)**	24 (8.8)	15 (12.5)	0.252	25 (10.2)	11 (9.4)	0.811
**CD4 <350**	90 (32.8)	49 (40.8)	0.127	84 (34.3)	42 (35.9)	0.763
**PVL, mean (SD), log_10_ copies/ml**	2.98 (1.5)	3.07 (1.5)	0.982	3.04 (1.5)	3.03 (1.5)	0.920
**PVL <400 copies/ml, n (%)**	127 (46.4)	59 (49.2)	0.606	126 (51.4)	61 (52.1)	0.9
**CART, n (%)**	179 (65.3)	75 (62.5)	0.589	154 (62.9)	74 (63.2)	0.943
**History of prior syphilis, n(%)**	105 (38.3)	61 (50.8)	0.021	94 (38.4)	57 (48.7)	0.062

**Abbreviations**: cART, combination antiretroviral therapy; MSM, men who have sex with men; SD, standard deviation.

In multivariate analysis, the only factor that was statistically significantly associated with serological response at 6 months of follow-up was age less than 34 years (adjusted odds ratio [OR], 1.96; 95% confidence interval [CI], 1.247–3.080; *P* = 0.004), while the associations between secondary syphilis (adjusted OR, 1.499; 95% CI, 0.943–2.383; *P* = 0.087) and CD4 <350 cells/µl (adjusted OR, 0.672; 95% CI, 0.425–1.065, *P* = 0.091) and serological response were of borderline significance (Table 3). At 12 months of treatment, patients with secondary syphilis were more likely to achieve serological response (adjusted OR, 1.938; 95% CI, 1.21–3.098, *P* = 0.006), while those with early latent syphilis were less likely to achieve serological response (adjusted OR, 0.806; 95% CI, 0.686–0.947, *P* = 0.009). The treatment regimen was not associated with serological response either at 6 months (adjusted OR, 1.315; 95% CI, 0.808–2.318, *P* = 0.270) or 12 months (adjusted OR, 0.919; 95% CI, 0.535–1.578, *P* = 0.759) of follow-up (Table 3).

**Table 3:** Multivariate analysis of factors associated with serological response at 6 and 12 months following treatment of early syphilis.

	6 months			12 months		
Variable	AOR	95% CI	*P*-value	AOR	95% CI	*P*-value
**Age <34 years**	1.96	1.247–3.080	0.004	1.268	0.795–2.024	0.319
**CD4 <350 cells/µl**	0.672	0.425–1.065	0.091	0.893	0.556–1.433	0.639
**Prior syphilis**	0.769	0.477–1.241	0.282	0.801	0.489–1.313	0.379
**Secondary syphilis**	1.499	0.943–2.383	0.087	1.938	1.212–3.098	0.006
**Early latent syphilis**	0.893	0.762–1.046	0.160	0.806	0.686–0.947	0.009
**Benzathine penicillin treatment**	1.315	0.808–2.318	0.270	0.919	0.535–1.578	0.759

**Abbreviations**: AOR, adjusted odds ratio; 95% CI, 95% confidence interval.

In the doxycycline group, secondary syphilis was the only factor that was statistically significantly associated with serological response both at 6 months (adjusted OR, 3.164; 95% CI, 1.326–7.553, *P* = 0.009) and 12 months (adjusted OR, 3.069; 95% CI, 1.096–8.594, *P* = 0.033) of treatment in the multivariate analysis (Table 4), while early latent syphilis was associated with failure to achieve serological response at 6 months (adjusted OR, 0.780; 95% CI, 0.598–1.017, *P* = 0.066) and 12 months (adjusted OR, 0.684; 95% CI, 0.496–0.944, *P* = 0.021) of follow-up.

**Table 4:** Multivariate analysis of factors associated with serological response at 6 and 12 months in the doxycycline group.

	6 months			12 months		
Variable	AOR	95% CI	*P*-value	AOR	95% CI	*P*-value
**Age <34 years**	1.988	0.884–4.470	0.097	1.365	0.501–3.718	0.543
**CD4 <350 cells/µl**	0.695	0.309–1.564	0.379	0.991	0.372–2.638	0.985
**Prior syphilis**	0.550	0.234–1.295	0.171	0.418	0.138–1.264	0.122
**Secondary syphilis**	3.164	1.326–7.553	0.009	3.069	1.096–8.594	0.033
**Early latent syphilis**	0.780	0.598–1.017	0.066	0.684	0.496–0.944	0.021

**Abbreviations**: AOR, adjusted odds ratio; 95% CI, 95% confidence interval.

Of the two treatment groups, 75 patients (27.7%) in the penicillin group and 45 patients (36.6%) in the doxycycline group were categorized as non-responders at 6 months after completion of treatment. The reasons for failure to achieve serological response at 6 months for the two groups are shown in Table 5. Of those non-responders, 3 patients (6.7%) were classified as having reinfection with syphilis (1 with secondary syphilis and 2 with 4-fold or greater rebound of RPR titers) and 42 patients (93.3%) as treatment failure in the doxycycline group; in the penicillin group, 15 patients (20.0%) were classified as having reinfection with syphilis (7 with primary or secondary syphilis and 8 with 4-fold or greater rebound of RPR titers) and 60 patients (80.0%) as treatment failure (Table 5).

**Table 5:** Non-responders at 6 months in the doxycycline and penicillin group who had completed 6 months of follow-up.

Non-responder at 6 months		Doxycycline N = 45	Penicillin N = 75
3-month follow-up (−)/6-month follow-up (−)	Reinfection§	0	0
	Treatment failure§§	6	15
3-month follow-up (−)/6-month follow-up (+)	Reinfection	1	2
	Treatment failure	9	13
3-month follow-up (+)/6-month follow-up (−)	Reinfection	1	1
	Treatment failure	13	11
3-month follow-up (+)/6-month follow-up (+)	Reinfection	1	12
	Treatment failure	14	21
	Total cases of reinfection, n (%)^a^	3 (6.7)	15 (20.0)
	Total cases of treatment failure, n (%)	42 (93.3)	60 (80.0)

Note: "3 month follow-up (−)" indicates patients had no follow-up of RPR titer at 3 months of treatment, while " 3 month follow-up (+)"indicates patients had follow-up of RPR titer; "6 month follow-up (−)" indicates patients had no follow-up of RPR titer at 6 months of treatment, while "6 month follow-up (+)" indicates patients had follow-up of RPR titer.

§ Re-infection: development of new symptoms of primary syphilis and secondary syphilis; or a 4-fold or greater increase of RPR titer after ever achievement of 4-fold or greater decline following treatment.

§§ Treatment failure: failure of RPR titer to decrease by 4 folds or greater, or receipt of another course of treatment without demonstrated serological response throughout the follow-up period.

a There was 1 case of secondary syphilis in the doxycycline group, while there were 2 cases of primary, 3 secondary, and 2 primary as well as secondary syphilis in the penicillin group.

Of the patients who had completed 12 months of follow-up after treatment, 86 and 31 patients in the penicillin and doxycycline group were categorized as non-responders. The reasons for failure to achieve serological response at 12 months for the two groups are shown in Table S1. Of those non-responders, 8 patients (25.8%) were classified as having reinfection with syphilis (1 with secondary syphilis and 7 with 4-fold or greater rebound of RPR titers) and 23 patients (74.2%) as treatment failure in the doxycycline group; in the penicillin group, 56 patients (65.1%) were classified as having reinfection with syphilis (23 with primary or secondary syphilis and 33 with 4-fold or greater rebound of RPR titers) and 30 patients (34.9%) as treatment failure (Table S1).

## Discussion

In this multicenter, retrospective observational study among HIV-infected patients with early syphilis, the serological response rate to a 2-week course of doxycycline (65.9%) was similar to that to a single dose of benzathine penicillin (68.3%) at 12 months of follow-up, though the response rate to doxycycline appeared to be lower than that to benzathine penicillin at 6 months of follow-up. In multivariate analysis, we found that secondary syphilis, but not treatment regimen, was associated with serological response at 6 and 12 months of follow-up.

While statistically significant difference in terms of serological response to benzathine penicillin versus doxycycline was not demonstrated in this study, the response rates to doxycycline at 6 and 12 months of follow-up are lower compared with those reported in the previous studies [17], [18]. The discrepancy may be explained by the study population, stage of syphilis of the patients, and definition of serological response. In our study, all of our patients with early syphilis had HIV infection and more than 90% of them were MSM who were treated with doxycycline, which is higher than those in previous studies in which HIV-infected patients accounted for 11% to 80% of the patients enrolled [17], [19]. HIV-infected patients and MSM with syphilis have been observed to have a higher risk for serological failure than HIV-uninfected patients [23]. According to the study by Ghanem et al [13], HIV-infected patients had a 6-fold higher risk of serological failure than HIV-uninfected patients. In the study by Psomas et al in which 80.2% of the patients had HIV infection, the overall serological response to doxycycline was 73.3% [19]; however, only 15 patients received doxycycline and the case number of HIV-infected patients who received doxycycline in the study by Psomas et al is not clearly described, which precludes us from estimating the serological response to doxycycline among the HIV-infected patients enrolled.

Treatment response of syphilis may be affected by many other factors such as RPR titer and stage of syphilis [23]–[25]. In the study of 156 HIV-infected patients with all stages of syphilis by Kim et al in an HIV outpatient clinic in New York City [24], a high RPR titer (>1∶64) and early stage of syphilis were significantly associated with treatment failure or reinfection, for which recurrent exposure to syphilis in the sexual network and reinfection have been proposed. In our study, the proportion of RPR titer of 64 or higher was 58.5% in the doxycycline group and 68.3% in the penicillin group, both of which were greater than those reported in previous studies [17], [18]. In contrast, we found in this study that higher RPR titers were independently associated with serological response at 6 months of treatment (per 1-log_2_ increase, adjusted OR, 1.237; 95% CI, 1.084–1.414, *P* = 0.002) in multivariate analysis that included age, CD4 count, a history of prior syphilis, and treatment (Table S2).

Compared with patients with primary and secondary syphilis, we found that patients with early latent syphilis were less likely to achieve serological response in multivariate analysis, which is consistent with the findings by Knaute et al and Wu et al [25], [26]. In our study, the percentage of patients with early latent syphilis was significantly higher in the doxycycline group (49.6%) than in the penicillin group (25.3%), which is also higher than that in the study by Ghanem et al (32.4%) [17] and Psomas et al (27.8%) [19]. The higher percentage of patients with latent syphilis may have contributed to the lower overall serological response rate observed in the doxycycline group than the penicillin group in our study.

In either group of patients included in this study, we found that nearly one third of the patients failed to achieve serological response at 6 or 12 months of treatment, which may be caused by reinfection with syphilis or relapse/treatment failure. With the use of clinical assessment and currently available molecular techniques [27], to differentiate reinfection from relapse or treatment failure may be difficult in those high-risk patients who are classified as non-responders by serological criteria, although development of symptoms of primary and secondary syphilis following receipt of recommended treatments are highly suggestive of reinfection. In our study, treatment failure accounted for different proportions of the non-responders who received either doxycycline or penicillin at 6 and 12 months of treatment (Table 5 and Table S1). However, limited by missing RPR data during the follow-up and lacking information on risky sexual contacts in this retrospective study, cases of reinfection may have been misclassified as treatment failure, especially in the patients who had reinfection but did not develop clinical symptoms or the patients sought clinical visit before the symptoms occurred, which may lead to underestimation of the effectiveness of either treatment regimen.

The strengths of our study are that our study has the largest case number of HIV-infected patients with early syphilis who were treated with doxycycline; patients with low RPR titer (1∶2 or undiluted only) were excluded to prevent difficulties in interpretation of the response or biologic false-positive reaction; the serological responses were determined at the recommended 6 and 12 months after treatment; and we had a large number of patients as a comparator group who received the recommended a single dose of benzathine penicillin for early syphilis and were assessed using the same definitions. There are several limitations in our study, however, and interpretation of our results should be cautious. First, this is a retrospective study and the follow-up blood testing was not performed in all patients at the recommended 3- to 6-month intervals after treatment was administered. Missing data that was treated with the last-observed-carried-forward principle may have caused underestimation of the serological responses to doxycycline. If we adopt the definition of serologic response used in Ghanem study (serological failure rate, lack of 4-fold drop within 270–400 days after treatment or a 4-fold increase within 30–400 days after therapy) [17], the response rate in our study will increase from 63.4% to 72.3% at 6 months in the doxycycline group (data not shown). Second, the case number for patients receiving doxycycline who had completed the 12 months of follow-up remains small and our study does not have sufficient power to confirm the non-inferiority of doxycycline to benzathine penicillin in treatment of early syphilis among HIV-infected patients. Third, our study did not include the patients with neurological, ophthmologic, or otic symptoms suggestive of neurosyphilis and the serological responses of such patients to doxycycline versus benzathine penicillin cannot be evaluated. Fourth, the follow-up duration of our study remains short and, therefore, we are not able to assess the effectiveness of benzathine penicillin or doxycycline in preventing neurological complications in this immunosuppressed population. Fifth, we were not able to ensure the adherence of the patients to the self-administered, 2-week course of doxycycline, while benzathine penicillin was administered intramuscularly at the hospital or clinic setting.

In conclusion, our multicenter, retrospective study demonstrated that the serological response rates to a 14-day course of doxycycline and a single dose of benzathine penicillin were similar in HIV-infected patients with early syphilis at 6 and 12 months of follow-up. Patients with secondary syphilis were more likely to achieve serological response than those with other stages. Our findings may provide support to the guidelines [15], [16] that doxycycline is an acceptable alternative agent for the treatment early syphilis among HIV-infected patients who are allergic to penicillins.

## Supporting Information

Table S1 Non-responders at 12 months in the doxycycline and penicillin group who had completed 12 months or longer follow-up.
(DOCX)

Table S2 Multivariate analysis of the factors associated with serological responses.
(DOCX)
