# Supplementary material for: Comparison of Serological Response to Doxycycline versus Benzathine Penicillin G in the Treatment of Early Syphilis in HIV-Infected Patients: A Multi-Center Observational Study
Source: PLoS One. 2014 Oct 13;9(10):e109813. doi: 10.1371/journal.pone.0109813 (PMC4195693; doi:10.1371/journal.pone.0109813)
Supplement: Table S1 — Non-responders at 12 months in the doxycycline and penicillin group who had completed 12 months or longer follow-up. (DOCX) [file pone.0109813.s001.docx]

**Table S1.** Non-responders at 12 months in the doxycycline and penicillin group who had completed 12 months or longer follow-up

| **Non-responder at 12 months** | | **Doxycycline**  **N = 31** | **Penicillin**  **N = 86** |
| --- | --- | --- | --- |
| 6month follow-up (-)/12month follow-up (-)^†^ | Reinfection^§^ | 2 | 5 |
|  | Treatment failure^§§^ | 10 | 6 |
| 6month follow-up (-)/12month follow-up (+) | Reinfection | 1 | 4 |
|  | Treatment failure | 1 | 4 |
| 6month follow-up (+)/12month follow-up (-) | Reinfection | 0 | 21 |
|  | Treatment failure | 5 | 8 |
| 6month follow-up (+)/12month follow-up (+) | Re-infection | 5 | 26 |
|  | Treatment failure | 7 | 12 |
|  | Total cases of reinfection, n (%)^a^ | 8 (25.8) | 56 (65.1) |
|  | Total cases of treatment failure, n (%) | 23 (74.2) | 30 (34.9) |

**Note:** "6 month follow-up (-)" indicates patients had no follow-up of RPR titer at 6 months of treatment, while "6 month follow-up (+)" indicates patients had follow-up of RPR titer; "12 month follow-up (-)" indicates patients had no follow-up of RPR titer at 12 months of treatment, while "12 month follow-up (+)" indicates patients had follow-up of RPR titer.

^§^Re-infection: development of new symptoms of primary syphilis and secondary syphilis; or a 4-fold or greater increase of RPR titer after ever achievement of 4-fold or greater decline following treatment

^§§^Treatment failure: failure of RPR titer to decrease by 4 folds or greater, or receipt of another course of treatment without demonstrated serological response throughout the follow-up period.

^a^ There were 1 cases of secondary syphilis in the doxycycline group, while there were 4 cases of primary, 16 secondary, and 3 primary as well as secondary syphilis in the penicillin group.
