# Supplementary material for: Comparison of Serological Response to Doxycycline versus Benzathine Penicillin G in the Treatment of Early Syphilis in HIV-Infected Patients: A Multi-Center Observational Study
Source: PLoS One. 2014 Oct 13;9(10):e109813. doi: 10.1371/journal.pone.0109813 (PMC4195693; doi:10.1371/journal.pone.0109813)
Supplement: Table S2 — Multivariate analysis of the factors associated with serological responses. (DOCX) [file pone.0109813.s002.docx]

**Table S2. Multivariate analysis of the factors associated with serological responses**

|  | Serological response at 6 months | | | Serological response at 12 months | | |
| --- | --- | --- | --- | --- | --- | --- |
| Variable | AOR | 95% CI | P-value | AOR | 95% CI | P-value |
| CD4 <350 cells/μl | 0.630 | 0.323-1.228 | 0.174 | 0.742 | 0.382-1.439 | 0.377 |
| Age <34 years | 1.978 | 1.249-3.133 | 0.004 | 1.349 | 0.845-2.153 | 0.210 |
| Prior syphilis | 0.615 | 0.378-1.000 | 0.050 | 0.670 | 0.410-1.096 | 0.111 |
| RPR titer, per 1-log_2_ increment | 1.237 | 1.084-1.411 | 0.002 | 1.096 | 0.960-1.250 | 0.174 |
| Penicillin treatment | 1.282 | 0.784-2.099 | 0.322 | 0.962 | 0.561-1.650 | 0.888 |
| CD4 count when treatment was administered | 1.000 | 0.998-1.001 | 0.782 | 0.999 | 0.998-1.001 | 0.432 |

**Abbreviations:** 95% CI, 95% confidence interval; AOR, adjusted odds ratio; RPR, rapid plasma reagin
